# Weathering the Storm: Thyroid Storm Precipitated by Radioiodine Contrast in Metastatic Thyroid Carcinoma

**DOI:** 10.7759/cureus.14219

**Published:** 2021-03-31

**Authors:** Brinda Basida, Nirav Zalavadiya, Rana Ismail, Hicham Krayem

**Affiliations:** 1 Department of Internal Medicine, Detroit Medical Center Sinai-Grace Hospital, Detroit, USA; 2 Department of Pulmonary and Critical Care Medicine, Detroit Medical Center Sinai-Grace Hospital, Detroit, USA

**Keywords:** thyrotoxicosis, metastatic thyroid cancer, jod-basedow phenomenon

## Abstract

Thyroid storm is an extremely rare yet life-threatening medical emergency. It results from the decompensation of undiagnosed or undertreated hyperthyroidism in the presence of an acute stressor such as trauma to the thyroid, infections, acute iodine load, withdrawal from the antithyroid medication, or surgical procedures (including thyroid surgery). Clinical features of thyroid storm include hyperthermia, tachycardia, respiratory distress, gastrointestinal and hepatic symptoms, and central nervous system dysfunction. It is primarily a clinical diagnosis, further aided by abnormal thyroid function tests. Thyroid storm is associated with significant mortality and morbidity - the latter mostly related to complications from thyrotoxicosis or hyperthyroidism. Treatment with iodine (or iodide-ionized active form of iodine) supplements or with radioactive iodine, also known as radioiodine, such as in the treatment of thyroid cancer, is a common and mostly safe practice; however, iodine contrast in tomography imaging may precipitate a thyroid storm in sporadic cases. Here, we report a remarkable case of a 62-year-old African American female with a history of total thyroidectomy secondary to follicular thyroid cancer three years before the current presentation; she developed left lung pneumonia complicated by thyroid storm status post a computed tomography angiogram of the abdomen. She exhibited signs and symptoms of thyrotoxicosis a few days after receiving the iodinated contrast. The recommended daily iodide intake for adults with hyperthyroidism is about 150 mcg per day, while a computed tomography scan exposes patients to 14 to 35 million mcg of iodinated contrast at once, which could have triggered a storm. In this case, the patient was diagnosed with thyroid storm, which was presumed to be a consequence of the Jod-Basedow phenomenon secondary to metastatic thyroid carcinoma lesions discovered later. This clinical diagnosis was reinforced by laboratory results showing elevated serum free T4 and undetectable thyroid-stimulating hormone. She was treated with supportive measures, steroids, beta-blockers, and antithyroid medications with a positive outcome. This case demonstrated that, in the setting of recurrent metastatic thyroid cancer, clinicians should approach the use of intravenous iodine medium contrast in imaging with some level of caution when dealing with patients at risk of thyrotoxicosis or with underlying hyperthyroidism state at the brink of a storm.

## Introduction

Thyroid storm, also called thyrotoxic crisis, is one of the most dreaded diagnostic challenges in clinical medicine. It is postulated that thyroid storm could be due to a rapid rise in serum thyroid hormone levels, enhanced responsiveness to catecholamines, or elevated cellular responses to thyroid hormone [1]. Most of the epidemiological data on thyroid storm comes from national surveys from the United States and Japan. Japan Thyroid Association reported a thyroid storm incidence of 0.2 cases/100,000 persons per year [2,3] compared to an incidence of 0.57-0.76 cases/100,000 persons per year in the United States based on 10-year inpatient data [4]. The Japanese surveys reported a thyroid storm incidence of 6.3 cases per 100,000 hospitalized patients per year [5] compared to the US national database that revealed 4.8 to 5.6 cases per 100,000 hospitalized per year [4]. Findings from the most extensive Japanese inpatient database with 21 million hospitalized patient data collected between 2011 and 2014 reported a 10.1% mortality rate among the 1324 hospitalized thyroid storm cases [5], and 10.7% in the other Japanese survey [2], and compared to a mortality rate between 1.2% and 3.6% among the hospitalized in the 10-year US study [4].

Given the severity of the thyroid storm condition, it is essential to mention some of the factors that precipitate its development, such as trauma to the thyroid gland, infection, thyroid cancer, Graves' disease, antithyroid medication non-use/non-compliance, and acute excessive iodine load [4]. The role of iodine in thyroid health is well-known, as it is implicated in controlling the overall thyroid gland function through the synthesis and regulation of thyroid hormones, the triiodothyronine (T3) and the thyroxine (T4), in collaboration with the pituitary gland that secretes the thyroid-stimulating hormone (TSH or thyrotropin). TSH works by the uptake of iodine to enhance the production of T3 and T4. In low iodine intake, the patient may have a high TSH level which causes follicular hyperplasia and eventually thyroid enlargement. In severe iodine deficiency, TSH secretion increases but may not be accompanied by increased thyroid hormones [6]. Nevertheless, an excess intake of iodine over time increases TSH production; however, it might have a temporary inhibitive effect on the release of T3 and T4 into the circulation, exhibiting the Wolff-Chaikoff effect. This effect may be prolonged in patients with underlying thyroid disorders [6,7]. For instance, the acute excessive iodine intake may exhibit the Wolff-Chaikoff effect, and a high TSH might lead to follicular hyperplasia, nodular goiters, hypothyroidism, thyroid cancer, or iodine-induced thyrotoxicosis, which might end in a storm [6]. In some patients with or at risk of thyroid dysfunction, excess iodine may provoke the development of iodine-induced hyperthyroidism or Jod-Basedow disease [6,7].

The recommended daily iodide consumption for adults is about 150 mcg per day, while a computed tomography (CT) scan exposes patients to 14 to 35 million mcg of iodinated contrast [6,7]. The maximum tolerated dose in adults is 600-1000 mcg per day without significant side effects [6]. This case exemplifies a remarkable representation of thyroid storm that resulted from using intravenous iodinated contrast in a patient with a history of total thyroidectomy secondary to thyroid cancer.

Abstract for this was previously presented as a poster at the American College of Physicians (ACP) Michigan Chapter Fall Scientific Meeting 2020.

## Case presentation

**Figure 1:**
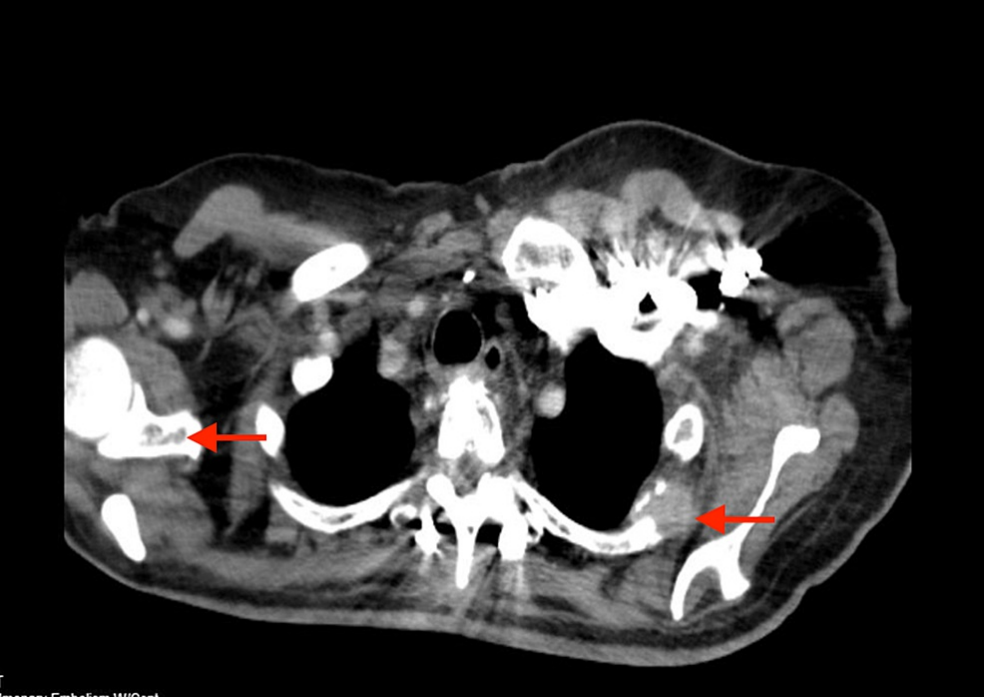
Computed tomography - pulmonary embolism of the thorax with contrast showing intraosseous metastasis (arrows) involving the left second rib and glenoid of the right scapula

An African American woman in her early 60s presented to the hospital with fever, vomiting, and abdominal pain. Her medical history was significant for non-obstructive coronary artery disease, chronic obstructive pulmonary disease on 2 L home oxygen, history of a pulmonary embolism on anticoagulation, and follicular thyroid cancer s/p total thyroidectomy three years ago (post-thyroidectomy labs: TSH 7.14 micro IU/mL, FT4 0.7 ng/dL). She did not take levothyroxine. The patient was afebrile, had tachycardia with a heart rate of 114 bpm, blood pressure 117/58 mmHg, respiratory rate 18/minute, and oxygen saturation at 96% with a 4 L nasal cannula. She was disoriented, had non-labored breathing with left infra-scapular crackles on auscultation and mild abdominal tenderness. Abdomen-pelvis CT with iodinated contrast showed stercoral colitis. Her chest x-ray revealed left lower lobe pneumonia. She completed five days of cefepime-metronidazole therapy and seven-day treatment with ertapenem for extended-spectrum beta-lactamase (ESBL)-producing *Escherichia coli *bacteremia. Following treatment, her clinical condition improved, and her vital signs stabilized to normal range. A thyroid function test prior to this admission showed TSH 0.89 micro IU/mL, free T4 1.25 ng/dL, and total T3 66 ng/dL. On day 5, the patient became extremely agitated and developed dyspnea and chest pain. She had fever, hypertension, and severe tachycardia with a heart rate in the 180-190s range. Electrocardiogram revealed supraventricular tachycardia (SVT) with no ST/T wave changes. Head CT without contrast was negative for intracranial abnormalities. CT angiogram of the thorax showed no pulmonary embolism but identified progressing metastasis in the left second and third ribs, right sixth rib, and glenoid of right scapula, right adrenal gland, and spleen (Figure 1). Arterial blood gases showed respiratory alkalosis with increased anion gap metabolic acidosis and elevated lactic acid of 6.1 mMol/L. She was temporarily started on IV heparin drip for elevated troponins of 0.57 ng/mL. Attempts at cardioversion with adenosine, diltiazem, and electric cardioversion were unsuccessful. Her hospital course was complicated with respiratory failure requiring laryngeal intubation due to worsening tachypnea and hypoxemia. Her tachycardia slightly improved with one to two doses of amiodarone and digoxin but did not resolve completely. Furthermore, her labs revealed severe hyperthyroidism with TSH <0.01 micro IU/mL, free T4 5.2 ng/dL, total T3 >395 ng/dL, total T4 > 30 g/dL, thyroglobulin 450 ng/mL, and thrombocytopenia. Lab results were negative for the anti-thyroid peroxidase and anti-thyroglobulin antibodies. Diagnosis of thyroid storm with a Burch-Wartofsky Point Scale (BWPS) score of 75 was made, thus, explaining the refractory SVTs. Amiodarone and digoxin were discontinued immediately, and she was started on propylthiouracil, hydrocortisone, metoprolol, and cholestyramine.

**Figure 2:**
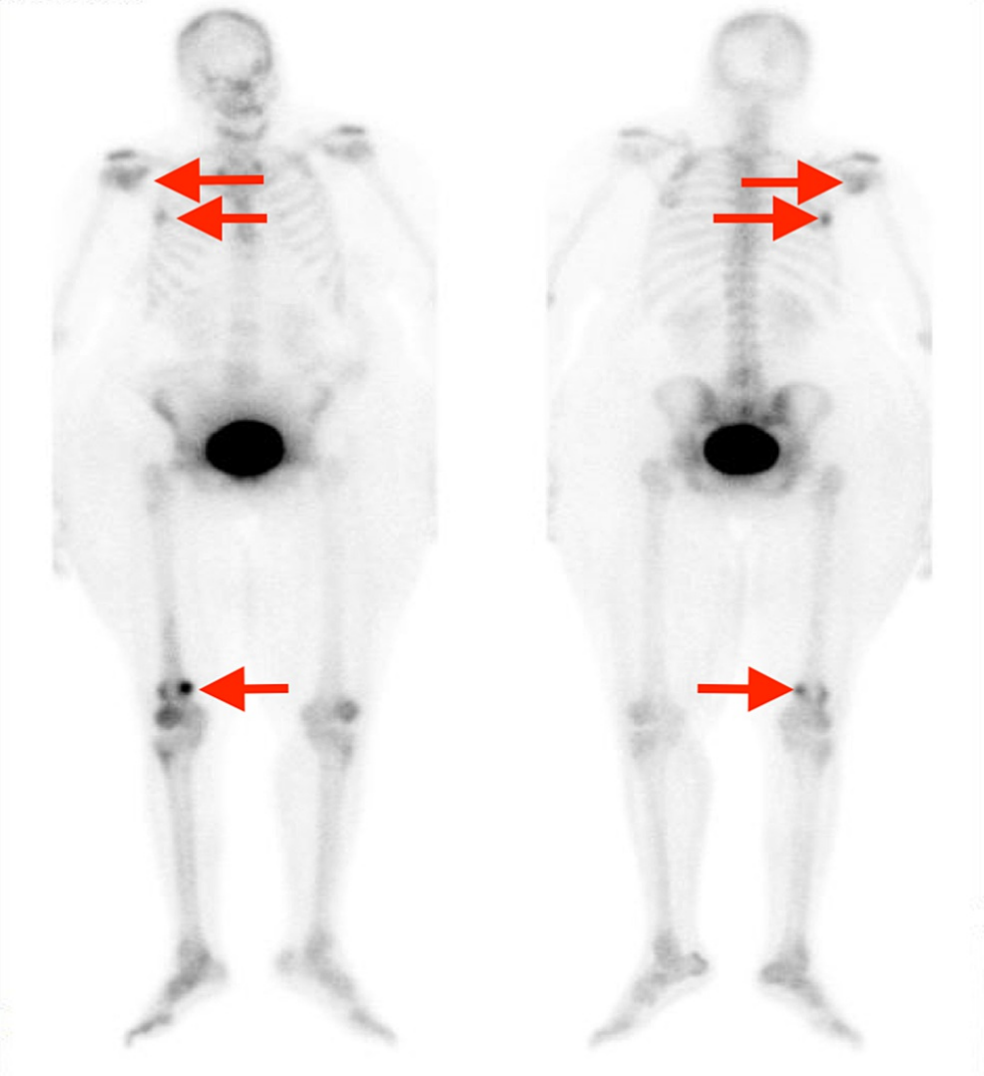
NM bone scan showing metastasis (arrows) in the right glenoid, right sixth rib, and right distal femur NM, nuclear medicine

Due to elevated liver function tests, propylthiouracil was replaced by methimazole. Following treatment for a couple of days, the patient's heart rate stabilized, and she improved clinically. Her mentation was back to baseline. Repeat thyroid function tests at week 6 from the initial presentation revealed free T4 levels at 1.01 ng/dL and normal TSH, thus validating the diagnosis of transient thyrotoxic crisis due to the Jod-Basedow phenomenon. The whole-body nuclear medicine (NM) bone scan showed multiple lesions in the right glenoid, on the right sixth rib, left second and third rib, and the right distal femur (Figure 2), suggestive of underlying metastatic disease. However, due to severe deconditioning, the patient could not tolerate liberation from mechanical ventilation and eventually underwent tracheostomy and percutaneous endoscopic gastrostomy tube placement. The patient was safely discharged to a nursing home for better transition of care and was periodically followed up and monitored for serum TSH and free T4 levels, which remained within normal limits. Eventually, she received several doses of radiation therapy for metastatic thyroid cancer.

## Discussion

The supportive findings for hormonally active metastatic thyroid cancer, in this case, are normal thyroid function tests in the absence of thyroid gland (TSH 0.89 micro IU/mL, free T4 1.25 ng/dL, total T3 66 ng/dL), elevated thyroid function tests in response to iodinated contrast in a patient with total thyroidectomy (TSH <0.01 micro IU/mL, free T4 5.2 ng/dL, total T3 >395 ng/dL), metastatic lesions identified on CT scan and NM bone scan, and resolution of symptoms and improving thyroid function test with antithyroid treatment. All these with a previous history of follicular thyroid cancer validate our diagnosis.

Hormonally active thyroid metastatic lesions are not common and have been described mostly with follicular thyroid carcinoma [8]. Other high-risk groups for iodine-induced thyrotoxicosis include multinodular goiter, Graves' disease, residing in an iodide-deficient area, and the elderly population [9]. In this patient with thyroidectomy, thyroid cancer metastasis was responsible for precipitating thyroid storm following iodine contrast administration since she had normal thyroid function tests before this hospitalization. This iodine-induced thyrotoxicosis was likely through the Jod-Basedow phenomenon [10], which led to hypersecretion of thyroid hormones in response to administering iodinated compounds in patients with underlying thyroid disease.

CT contrast can be an important, albeit unusual, precipitating factor for thyroid storm. The reported incidence of contrast-induced hyperthyroidism varies widely from 0.05% to 5% [11]. This induced hyperthyroidism develops variably from few days to 16 weeks following the administration of the iodinated contrast [7,12]. Torres et al. had presented a case of iodide-induced hyperthyroidism within four hours of iodine exposure; this was reported as one of the shortest courses between iodine contrast administration and symptoms manifestation [9].

Based on clinical criteria, a BWPS score of 45 is highly suggestive of thyroid storm (Table 1) [13]. A BWPS score of 75 with significant TSH suppression and marked elevation of free T4 and T3 provided strong evidence for thyroid storm diagnosis in our patient. Antithyroid drugs like propylthiouracil and methimazole effectively block thyroid hormone production by such functioning tumors [8]. In our patient, too, circulating thyroid hormone levels decreased after treatment with methimazole. In addition to antithyroid agents, glucocorticoids are also used to manage thyroid storm and help by reducing T4 to T3 conversion, promoting vasomotor stability, and possibly treating associated relative adrenal insufficiency [14]. Supportive therapy in an intensive care unit and management of any precipitating factors, along with specific therapy directed against the thyroid, are crucial due to the very high mortality rate of thyroid storm (10%-30%) [2,5,15-16].

**Table 1:** Burch-Wartofsky Point Scale Total points: <25, storm unlikely; 25-45, impending storm; >45, thyroid storm Source: [13]

Clinical characteristics	Score
Temperature (°F)	
99-99.9	5
100-100.9	10
101-101.9	15
102-102.9	20
103-103.9	25
104 or higher	30
Cardiovascular dysfunction	
Tachycardia (beats/min)	
99-109	5
110-119	10
120-129	15
130-139	20
140 or higher	25
Atrial fibrillation	10
Central nervous system effects	
Absent	0
Mild (agitation)	10
Moderate (delirium, psychosis, extreme lethargy)	20
Severe (seizure, coma)	30
Heart failure	
Mild (pedal edema)	5
Moderate (bibasilar rales)	10
Severe (pulmonary edema)	15
Gastrointestinal-hepatic dysfunction	
Moderate (diarrhea, nausea/vomiting, abdominal pain)	10
Severe (unexplained jaundice)	20
Precipitant history	
Positive	0
Negative	10

Due to the current growth in the utilization of iodinated contrast medium radiography, it is necessary to use prudent clinical judgment to prevent iodide-induced thyrotoxicosis in high-risk patients. Although prophylactic treatment with perchlorate and a thionamide class drug before iodine administration has shown some benefit in preventing hyperthyroidism in high-risk groups like those with metastatic thyroid disease, multinodular goiter, Graves' disease, residing in an iodide-deficient area, and elderly population [17,18], it is not routinely recommended. Patients should be monitored carefully after iodinated contrast studies for any cardiovascular instability [19,20]. However, in patients with normal thyroid, routine thyroid function monitoring is not recommended before iodinated contrast administration [20].

## Conclusions

This case illustrates a fascinating property of some types of thyroid cancer, with hormonally active metastatic recurrence. It underscores the importance of obtaining a detailed medical history to identify patients at risk of thyroid storm and avoid iodinated compounds in high-risk subjects, if possible. A low index of suspicion in such patients facilitates prompt recognition and adequate early management, which is a key for a better patient outcome. Routine follow-up with serum T4 and TSH should be emphasized in all thyroid cancer patients after thyroidectomy to minimize advanced metastasis complications. It is better to test T4 and TSH in these patients before exposure to any excessive iodine load, primarily through iodinated contrast.
